# BH3-only protein Noxa contributes to apoptotic control of stress-erythropoiesis

**DOI:** 10.1007/s10495-013-0890-y

**Published:** 2013-08-23

**Authors:** Felix M. Wensveen, Christian R. Geest, Sten F. W. M. Libregts, Ingrid A. M. Derks, Paul G. Ekert, Verena Labi, Andreas Villunger, Martijn A. Nolte, Eric Eldering

**Affiliations:** 1 Department of Experimental Immunology, Academic Medical Center, Meibergdreef 8, 1105 AZ Amsterdam, The Netherlands; 2 Division of Cell Signaling and Cell Death, The Walter and Eliza Hall Institute of Medical Research, 1G Royal Parade, Melbourne, VIC Australia; 3 Division of Developmental Immunology, BIOCENTER, Innsbruck Medical University, Innrain 80-82, 6020 Innsbruck, Austria; 4 Department of Hematopoiesis, Sanquin Research and Landsteiner Laboratory AMC/UvA, Amsterdam, The Netherlands

**Keywords:** Noxa, Erythropoiesis, Apoptosis, Erythropoietin, Mcl-1

## Abstract

Apoptosis plays an essential role in the control of erythropoiesis under normal and pathological conditions. However, the contribution of individual proteins within cell death signalling pathways remains poorly defined. Here, we investigated the role of the pro-apoptotic Bcl-2 family member Noxa in the regulation of erythropoiesis. We found that expression of Noxa is induced during erythroid differentiation of human and murine precursor cells. Using in vitro model systems for erythroid progenitors, we observed rapid induction of Noxa upon cytokine deprivation. Knockdown or deletion of Noxa conferred significant protection against apoptosis upon cytokine withdrawal. In vivo, Noxa deficiency did not affect hematological blood parameters or erythroid progenitor composition of bone marrow and spleen under steady-state conditions. In contrast, in a model of acute haemolytic anemia, Noxa-deficiency enhanced hematocrit recovery. Moreover, in a model of chronic inflammation-induced anemia, Noxa-ablation resulted in a dramatic increase of erythroblast expansion. Our data indicate that induction of Noxa in erythroid progenitors sets a survival threshold that limits expansion beyond the number of cells that can be sustained by the available cytokines, which becomes apparent under conditions of induced anemia.

## Introduction

Red blood cell (RBC) production is a strictly regulated process and homeostatic maintenance of the erythropoietic system requires a delicate balance between generation and destruction of erythrocytes [1]. Hematopoietic cytokines play a crucial role in regulating this homeostatic balance and lack of appropriate growth factor stimulation results in rapid progenitor cell death [2]. Erythropoietin (EPO) is a lineage-specific hematopoietic cytokine [3] which functions primarily as an erythroblast survival factor. Its anti-apoptotic actions have been proposed to involve the apoptotic regulators Bcl-XL and Bim [4], which are prominent members of the Bcl-2 mitochondrial pathway of apoptosis [5]. Bcl-XL belongs to the anti-apoptotic members of the Bcl-2 family, which bind and antagonise pro-apoptotic BH3-only proteins, such as Bim and Noxa.

Gene-targeting studies in mice have identified the mitochondrial pathway of apoptosis as an important regulatory mechanism during erythropoiesis [6]. Mice that lack Bcl-XL die in utero due to a failure of embryonic stem cells to generate viable mature erythocytes [7]. Reduced Bcl-XL expression in early erythroblasts results in ineffective erythropoiesis and anemia [7, 8]. Studies of Bim-deficient mice showed that prevention of Bim activation is a key component of survival signalling mediated by cytokines in hematopoietic progenitors. EPO protects erythroblasts from apoptosis by downregulation of Bim expression and enhanced ERK-mediated degradation of the Bim protein [9].

Unlike Bim, which potently engages all pro-survival Bcl-2 molecules [10], Noxa exerts its pro-apoptotic function mainly by neutralizing the pro-survival Bcl-2 protein Mcl-1 [11]. Previously, Mcl-1 has been shown to play a detrimental role in the survival of hematopoietic progenitors. Genetically modified mice in which Mcl-1 can be deleted in an inducible fashion in hematopoietic stem cells rapidly lost these cells after gene-deletion [12]. Unfortunately, the strong phenotype of Mcl-1 deficiency prevents detailed investigation of its role in cell specific functions. In contrast, Noxa is thought to ‘sensitize’ cells to apoptotic stimuli, rather than inducing cell death in itself [13]. Noxa deficiency therefore allows investigation of the role of cell death pathways in physiological processes via subtle alteration of cell death sensitivity [14], rather than complete abrogation of these pathways such as is the case for Bim or Mcl-1 deficiency.

Previously, we have identified Noxa as an important regulator of effector T and B cell expansion [14–16]. In response to antigen, T and B cells rapidly induce Noxa, which sets a survival threshold that depends on antigen-affinity. This survival threshold ensures that, when lymphocytes of various affinities expand and compete for nutrients and pro-survival cytokines, only high-affinity cells prevail and are allowed to contribute to the effector cell pool. Whether Noxa also mediates expansion capacity of other cells within the hematopoietic lineage such as erythroblasts is currently unknown. Interestingly, the pro-survival activities of EPO have been proposed to involve PI3-kinase, which is an important regulator of Mcl-1 protein levels [17]. Therefore, the Noxa/Mcl-1 axis may also represent an important regulatory mechanism of erythroblast expansion under conditions of limiting EPO-concentrations.

Hence, we here set out to investigate the role of Noxa in regulating basal erythropoiesis and its contribution to stress-erythropoiesis in vivo. We find that Noxa is induced both in human and murine eryhtroid precursors. In vitro, reduced Noxa levels conferred a significant survival advantage of erythroid precursors upon cytokine-deprivation. In vivo, Noxa-deficiency did not affect the erythroid compartment under homeostatic conditions, but resulted in greatly enhanced erythroblast expansion in a model of chronic inflammation-induced anemia. In a model for acute haemolytic anemia, Noxa-deficiency enhanced hematocrit recovery. Our findings provide new insights in the regulation of erythropoiesis during acute and chronic anemia and suggest an important role for the Noxa/Mcl-1 axis in these processes.

## Materials and methods

### Mice

C57BL/6 (B6) JAX^®^ mice were purchased from Charles River and kept as breeding colonies in our local animal facility. Only these mice, which were kept under identical conditions as our knock-out mice, were used as WT controls in our experiments, unless stated otherwise. Noxa^−/−^ mice were a kind gift from Dr. A. Strasser (WEHI, Melbourne) and provided by Dr. M. Serrano (CNIO, Madrid). Bim^−/−^ mice, WT and Noxa^−/−^ controls for colony formation experiments were bred and housed in the central animal facility of the Innsbruck Medical University. CD70^TG^ mice were generated as described previously [18]. Mice were used at 6–12 weeks of age, unless stated otherwise, age- and sex-matched within experiments and were handled in accordance with institutional and national guidelines. All mice were either generated in B6 mice or backcrossed at least ten times on this background.

### Culture

Mononuclear cells were isolated from peripheral blood by density centrifugation over a Ficoll-Paque solution (density 1.077 g/mL). Immunomagnetic cell separation (Miltenyi Biotec) was used to isolate CD34+ cells. Erythrocytes were generated using a modified two-phase culturing system as described by Giarratana et al. [19]. Briefly, CD34+ cells were cultured in IMDM supplemented with 1 % bovine serum albumin (BSA), 120 g/mL iron-saturated human transferrin, 900 ng/mL ferrous sulfate, 90 ng/mL ferric nitrate, and 10 g/mL insulin (Sigma-Aldrich GmbH). Erythrocyte differentiation was induced on the addition of 100 ng/mL stem cell factor (SCF) (R&D systems), 10^−6^ M hydrocortisone (Sigma-Aldrich GmbH), 5 ng/mL interleukin-3 (IL-3) (R&D systems), and 3 IU/mL EPO (Aranesp^®^). After 8 days of culture, erythroblasts were cultured in the presence of EPO until day 14.

TF-1, FDM-WT and FDM-Noxa^−/−^ cells were generated as described previously [20] and maintained in RPMI (Invitrogen supplemented with 10 % fetal bovine serum (PAA Laboratories GmbH), 100 μg/mL Gentamycin (Invitrogen), 2 mM l-Glutamine (2 mM), 0,5 mM β-mercapto-ethanol (Sigma-Aldrich GmbH), and 0.1 ng/mL human recombinant IL-3 (R&D systems) at 37 °C and 5 % CO2.

### Lentiviral-mediated shRNA targeting

Lentiviral shRNA clones (Sigma Mission RNAi) targeting Noxa and a scrambled non-targeting control (SHC002) were purchased from Sigma. These vectors were co-transfected with the packaging vectors psMD2G, pMDLg/pRRE and pRSV-Rev into 293T cells using Fugene6 (Roche Applied Science) to produce the virus. After 48 h, lentiviral vector particles were harvested, virus supernatants were filtered with 0.22-μm filters and stored at −80 °C. Efficiency of different lentiviral shRNA clones in cells was determined by Western blot. TF-1 cells were transduced by a single round infection for 24 h using a combination of 5 different Sigma Mission RNAi clones, followed by puromycin (Sigma-Aldrich GmbH) selection (1 μg/mL) for 1 week.

### Measurement of apoptosis

Apoptotic cells were measured by staining with Annexin V-FITC (IQ Products, Groningen, The Netherlands) according to the manufacturer’s protocol for 20 min. Before analysis of the samples by FACS, propidium iodide (PI) (Sigma-Aldrich GmbH) was added (5 μg/mL). Viable cells were defined by Annexin V^−^/PI^−^ staining. Alternatively, cells were incubated with 200 nM Mitotracker (Molecular Probes) [21] for 30 min at 37 °C, washed, and double stained with Annexin V-APC. Viable cells were defined as Mitotracker ^hi^/Annexin V^−^.

### Western blot analysis

Western blot analysis was performed using standard techniques. In brief, cells were lysed in Laemmli buffer (0.12 M Tris–HCl pH 6.8, 4 % SDS, 20 % glycerol, 0.05 μg/μL bromophenol blue, and 50 mM Dithiothreitol) and boiled for 5 min. Protein contents were determined by the Bio-Rad protein assay (Bio-Rad Laboratories, Munchen, Germany) and equal amounts of total lysate were analyzed by 12 % SDS–polyacrylamide gel electrophoresis. Proteins were transferred to Immobilon-P and incubated with Tris buffered saline (TBS) containing 2 % low-fat milk for 1 h before incubating with an antibody against Noxa (Imgenex, San Diego, CA), Bcl-XL (Transduction Laboratories, Lexington, KY), Bim (Stressgen Bioreagents Canada), Puma (Cell Signaling), Bcl-2 (Alexis Biochemicals) or β-actin (Santa Cruz Biotechnology, Santa Cruz, CA) overnight at 4 °C in TBS-Tween. Blots were subsequently incubated with IRDye 680 or 800 labelled secondary antibodies for 1 h. Odyssey Imager (Li-Cor) was used as a detection method according to the manufacturer’s protocol.

### Flow cytometry and histology

Murine single-cell suspensions were obtained by mincing the specified organs through 40 μm cell strainers (Becton–Dickinson). Whole blood and tissue homogenate cell counts were measured on heparinized blood using an automated cell counter (Beckman Coulter). Cells (5 × 10^5^ to  5 × 10^6^) were collected in staining buffer (PBS with 0.5 % BSA) and stained for 30 min at 4 °C with antibodies against Ter119 (Ter119), CD71 (R17217) and CD117 (2B8). For FACS analysis of reticulocytes, 3–5 μL of heparinized blood was washed in staining buffer and stained for CD71. Cells were washed and resuspended in staining buffer, after which Thiazole Orange (Sigma-Aldrich GmbH) was added to a final concentration of 1 ng/mL and immediately analyzed. FACS experiments were performed on a FACSCalibur or FACSCanto (Becton–Dickinson) and analysed with FlowJo software (TriStar). All FACS antibodies were obtained from eBioscience. Standard haematoxylin and eosin staining was performed on PFA fixed, paraffin embedded tissues. Pictures were taken using a Leica microscope.

### RT-MLPA (Reverse transcriptase multiplex ligation-dependent amplification)

Total RNA for Reverse Transcriptase Multiplex Ligation-dependent Probe Amplification (RT-MLPA) was extracted using the Trizol isolation method (Invitrogen). mRNA levels were analyzed with the SALSA RT-MLPA Apoptosis kit R011-C1 (MRC-Holland, http://www.mlpa.com) according to the manufacturer’s instructions. Samples were run through a Genescan and analyzed with GeneMapper (Applied Biosystems GmbH; http://www.appliedbiosystems.com) and subsequently with Excel software (Microsoft), as described previously [22, 23].

### EPO ELISA and anemia induction

Serum EPO levels of mice were quantified by using the Quantikine Mouse/Rat EPO Immunoassay (R&D Systems) as described by the manufacturer. Acute anemia was induced by a single i.p. injection of 60 mg/kg phenylhydrazine (Sigma-Aldrich GmbH).

### Methylcellulose cultures

To determine colony forming unit-erythroid (CFU-e) and burst forming unit erythroid (BFU-e) potential, 3.75 × 10^5^ total bone marrow or 7.5 × 10^5^ total spleen cells were resuspended into 325 μL Iscove’s Modified Dulbecco’s Medium (IMDM). Cells were subsequently resuspended in 3.2 mL methylcellulose medium (MethoCult M3234, StemCell Technologies) supplemented with 4U mL human recombinant EPO (Aranesp^®^), 100 ng/mL murine recombinant SCF, 20 μg/mL iron-saturated human transferrin, 2 × 10^−4^ M hemin (Sigma-Aldrich), 1 % gentamycin and plated in triplo. CFU-e colonies were counted on day 3, BFU-e colonies on day 8 after plating.

### Statistical analysis

Statistical analysis was performed using the unpaired Student’s *t* test, Wilcoxon rank-sum test or one-way ANOVA test where applicable. Asterisks denote significant differences (*p < 0.05, **p < 0.005, ***p < 0.0005).

## Results

### Induction of Noxa in human hematopoietic progenitors during erythroid development

**Fig. 1:**
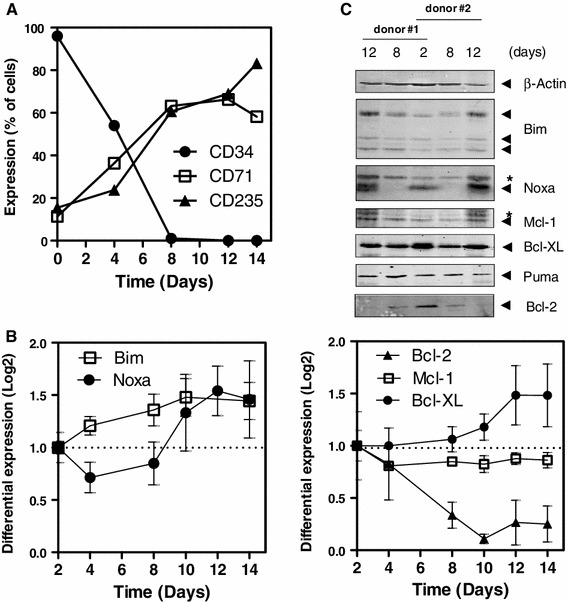
Noxa is induced in human hematopoietic progenitors during early erythroid development. **a** Purified CD34^+^ progenitors from peripheral blood were differentiated towards erythroblasts in 14 days. FACS analysis of cell cultures shows erythroid differentiation as exemplified by acquisition of CD71 and CD235a and loss of the stem cell marker CD34 in one representative experiment of 4 performed. **b** Expression profiling by RT-MLPA, reveals induction of Noxa, Bim and Bcl-XL and downregulation of Bcl-2 during erythroid development. Average of 4 independent experiments is shown, expressed as log transformed expression data in relation to day 2 of culture. The number of cells and the yield of RNA at day 0 was too low to allow reproducible analysis. **c** Protein lysates were prepared from CD34^+^ cells undergoing erythroid differentiation for 2, 8 and 12 days. Western blot analysis was performed using antibodies against Noxa, Bim, Puma, Bcl-XL, Mcl-1 and Bcl-2. β-Actin is used as loading control. Similar results were obtained in two independent experiments. To compensate for low cell numbers, protein lysates on day 2 were pooled. Asterisks mark non-specific bands. *Error bars* represent SEM

To explore the contribution of cell death regulators in the erythroid system, we analyzed the expression levels of Bcl-2 family members at different stages of erythroid development utilizing an ex vivo differentiation system. For this purpose, human CD34^+^ hematopoietic progenitors, isolated from peripheral blood, were cultured in presence of EPO, IL-3, SCF and hydrocortisone to induce erythrocyte differentiation. After 8 and 12 days, the majority of cells was characterized as erythroblast on the basis of morphology (data not shown), loss of CD34 expression and increased expression of erythrocyte markers CD71 and CD235a (Fig. 1a). At different time points, total mRNA was extracted from cells undergoing differentiation and expression levels of pro- and anti-apoptotic molecules was analyzed by RT-MLPA [23] (Fig. 1b). Of the BH3-only molecules, Noxa expression was downregulated during early stages of erythroid differentiation but increased as the majority of cells reached the erythroblast stage after day 8. Bim levels, on the other hand, steadily increased over time and reached a maximum on day 8. Analysis of pro-survival Bcl-2 family members revealed that human CD34^+^ hematopoietic cells express Bcl-2 at relatively high levels, which decline as cells undergo erythroid differentiation. Conversely, Bcl-XL is induced during differentiation towards the erythroid lineage. mRNA levels of Mcl-1 and Bfl-1/A1 remained relatively unchanged during differentiation (Fig. 1b, right panel and data not shown).

Since Noxa and its pro-survival antagonist Mcl-1 are highly regulated on a post-transcriptional level [17], Western blot analysis was performed to investigate whether the mRNA levels of these genes corresponded with protein expression (Fig. 1c). Noxa protein correlated closely with the fluctuations in its mRNA transcripts. Notably, changes in Noxa mRNA expression preceded changes on a protein level. Mcl-1 protein corresponded less well with its mRNA levels. Mcl-1 protein followed a similar expression level as Noxa, suggesting a role for these binding partners in the regulation of erythropoiesis.

### Noxa regulates survival of erythroid progenitors under cytokine limiting conditions

To study the correlation between Noxa expression and cell viability of erythroid cells, TF-1 cells were used as a defined cell line model for the regulation of erythropoiesis. TF-1 cells are committed early erythroid CD34^+^ cells, which are maintained in undifferentiated state in the presence of IL-3 or GM-CSF [24]. When cultured with EPO, these cells differentiate and obtain a phenotype that resembles that of erythroid precursors.

**Fig. 2:**
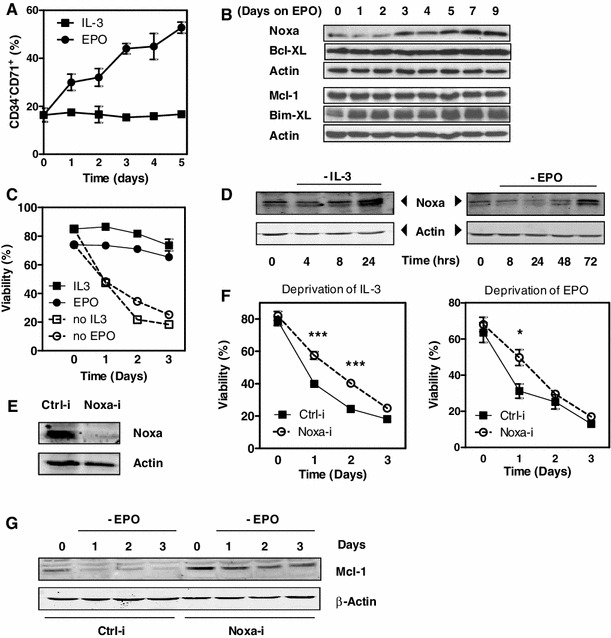
Noxa regulates survival of erythroid progenitors under cytokine limiting conditions. **a** TF-1 cells were cultured either in presence of IL-3 or EPO. Expression of CD34 and CD71 was analyzed by flow cytometry. The percentage of CD34^−^CD71^+^ cells is depicted. **b** TF-1 cells were cultured in the presence of EPO. At the indicated days, protein lysates were analysed using antibodies against Noxa, Bcl-XL, Mcl-1 and Bim-XL. Shown are samples of a single experiment, loaded on two separate gels, each with their own β-actin loading control. **c** TF-1 cells were cultured either in presence of IL-3 or EPO. After 4 days, cells were deprived of EPO or IL-3 and viability was determined by Annexin V/PI staining. One of 4 experiments is shown. **d** TF-1 cells were cultured either in presence of IL-3 (*left*) or EPO (*right*). After 4 days of culture, cells were deprived of EPO or IL-3 for indicated times and Noxa protein levels were determined by western blot. β-Actin was used as loading control. **e** Western blot showing expression of Noxa and Actin after knockdown of Noxa in TF-1 cells using a lentiviral vector containing a shRNA targeting Noxa (Noxa-i) or a scrambled non-targeting control (Ctrl-i). Noxa expression was induced upon culture in presence of 5 μM Bortezomib for 4 h prior to lysis. **f** Noxa-i or Ctrl-i cells were cultured with IL-3 or EPO for 4 days and subsequently deprived of either IL-3 or EPO for 72 h. Shown is viability, measured by Annexin V/PI staining, from the start of cytokine deprivation. Averages are shown of 9 independent experiments. **g** Noxa-i and Ctrl-i cells were cultured in presence of EPO for 4 days and subsequently deprived of this cytokine. After the indicated days, Mcl-1 protein levels were determined. β-Actin was used as a loading control. One of three experiments is shown. *Error bars* represent SEM

TF-1 cells were cultured in presence of EPO and their erythroid phenotype was characterized. Upon differentiation towards the RBC lineage, CD34 was downregulated and expression of the transferrin receptor (CD71) was induced (Fig. 2a). At protein level, Mcl-1 and Bcl-XL were highly expressed in this transformed cell line, and displayed no changes after EPO treatment (Fig. 2b). Bim was rapidly induced in presence of EPO, whereas Noxa expression was upregulated from day three onwards. These findings correspond well with our observations in primary human cells (Fig. 1).

To study responses to cytokine deprivation, TF-1 cells were cultured for 4 days with either IL-3 or EPO and subsequently deprived of cytokine for 24, 48 and 72 h. Viability was assessed by staining for Annexin V/PI. Following withdrawal of IL-3 or deprivation of EPO, TF-1 cells underwent rapid apoptosis, which corresponded with induction of Noxa protein (Fig. 2c, d). To investigate whether Noxa contributed to cytokine deprivation-induced cell death in TF-1 cells, a short hairpin RNA (shRNA) approach was used to reduce Noxa levels in TF-1 cells (Fig. 2e). Indeed, knockdown of Noxa conferred substantial protection from apoptosis induced by either IL-3 or EPO deprivation (Fig. 2f).

Finally, we investigated whether Noxa mediates its pro-survival effect via regulation of Mcl-1 protein levels. Upon binding, Noxa can target Mcl-1 for proteasomal degradation [11]. Therefore, we monitored Mcl-1 protein levels in EPO-differentiated TF-1 cells, and observed that Mcl-1 levels rapidly declined upon EPO-deprivation (Fig. 2g). Moreover, cells in which Noxa was knocked down had significantly higher levels of Mcl-1 under homeostatic conditions, which remained higher after deprivation of EPO.

Together, these data show that Noxa is involved in regulating apoptosis of hematopoietic progenitor cells under cytokine limiting conditions via control of Mcl-1 protein levels.

### Noxa regulates survival of murine erythroid progenitors under cytokine limiting conditions

**Fig. 3:**
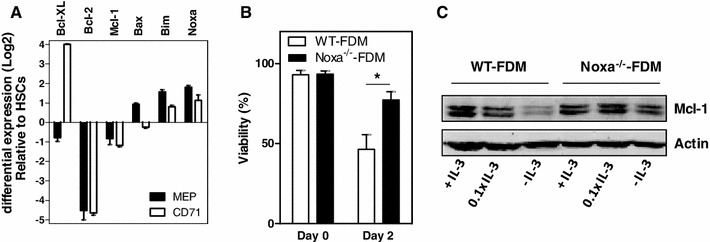
Noxa regulates survival of murine erythroid progenitor cells under cytokine limiting conditions. **a** Relative gene expression of sorted bone marrow derived murine MEPs (Lin^−^CD117^+^CD34^−^CD16^−^) and splenic CD71^+^ cells, compared to the expression in HSC/CMPs (Lin^−^CD117^+^CD34^+^CD16^Dim^), measured by RT-MLPA. Data is expressed on a logarithmic scale. *Error bars* represent SEM (*n* = 3). **b** FDM cells generated from WT or Noxa^−/−^ mice were deprived of IL-3 for 2 days. Viability was determined by staining for sub-G1 DNA containing cells. Data represent the mean of 5 experiments. *Error bars* represent SEM. **c** Western blot analysis of Mcl-1 levels in WT- or Noxa^−/−^-FDM cells cultured for 2 days in the presence of the minimal IL-3 concentration that sustained full proliferation and survival (+IL-3), 10 % of that concentration (0.1 × IL-3) or in absence of IL-3 (−IL-3). β-Actin is used as a control for equal loading

In order to investigate the contribution of Noxa to erythropoiesis in vivo, we first determined whether Noxa regulation is similar in mice and human erythropoiesis. Megakaryocyte/erythrocyte precursors (MEP) and more differentiated CD71^+^ erythroblasts were purified and mRNA levels of Bcl-2 family members was compared with that of early hematopoietic precursors. Similar to human erythropoiesis, Bcl-2 was downregulated, whereas Bcl-XL was strongly induced in erythroblasts (Fig. 3a). In addition, Bim and Noxa transcript levels increased during erythroid differentiation, whereas Mcl-1 levels slightly decreased. Thus, Noxa mRNA appears to follow a similar expression pattern in murine and human erythroid development. Due to lack of reliable antibodies for murine Noxa, we were unable to monitor protein levels.

These findings prompted us to investigate whether Noxa also mediates cell death upon cytokine deprivation of murine hematopoietic precursors. Since there are no murine pre-erythroid cell lines available with similar characteristics as TF-1 cells, we used IL-3 dependent cells (factor-dependent myeloid—FDM) generated from WT mice or animals lacking Noxa (Noxa^−/−^) [20]. WT- and Noxa^−/−^-FDM cells were deprived of IL-3 for 48 h and viability was assessed. Loss of Noxa in FDM cells resulted in increased resistance to apoptosis following IL-3 deprivation compared to WT-FDM cells (Fig. 3b). Similar to EPO-deprived TF-1 cells, Mcl-1 protein levels rapidly declined in FDM cells when IL-3 was removed, in a concentration-dependent fashion. In Noxa^−/−^-FDM cells, decrease of Mcl-1 protein was notably less upon IL-3 deprivation, compared to WT-FDM cells (Fig. 3c).

These findings indicate that also in mouse hematopoietic precursors, Noxa mediates survival via regulation of Mcl-1 protein levels upon cytokine deprivation.

### Noxa deficiency under homeostatic conditions results in mild anisocytosis in vivo

**Fig. 4:**
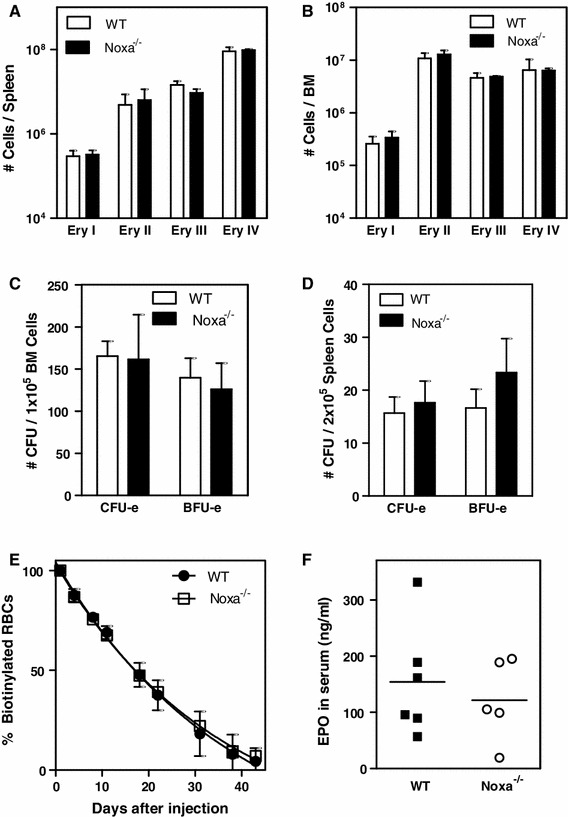
Noxa-deficiency does not affect erythropoiesis under homeostatic conditions. **a**–**b** Absolute numbers of erythroblast subsets in **a** spleen and **b** bone marrow of WT and Noxa^−/−^ mice. **c**–**d** Number of CFU-e and BFU-e colonies after 3 and 8 days of culture respectively in semi-solid methylcellulose containing erythropoiesis-promoting factors, using **c** 1 × 10^5^ Bone marrow or **d** 2 × 10^5^ spleen cells of WT and Noxa^−/−^ mice. Data show mean values ±SEM of at least 4 independent experiments with 3 mice in each experiment. **e** WT and Noxa^−/−^ mice were injected with active biotin. Percentage of biotinylated red blood cells was followed over time by FACS. Data show mean values ± SEM of an experiment using 3 mice per group. **f** EPO levels (ng/mL) in the serum of WT and Noxa^−/−^ mice, as determined by ELISA

Having established the effects of Noxa on cell viability of erythroid cells in vitro, we next determined the physiological impact of Noxa-ablation on erythroid development in vivo. Erythroblast subsets [8] were quantified in spleen and bone marrow (BM) of WT and Noxa^−/−^ mice [25], but no differences were observed in either erythropoietic organ (Fig. 4a, b). To assess the clonogenic capacity of WT and Noxa^−/−^ spleen- and BM-resident erythroid progenitors, the number of CFU-e and BFU-e colonies was determined after 3 and 8 days of culture respectively (Fig. 4c, d). Noxa deletion did not significantly affect the colony forming capacity of either bone marrow- or spleen-derived progenitors. Furthermore, hematological blood parameters, including RBC numbers, hemoglobin levels, and hematocrit appeared normal when compared to syngeneic control mice (Table 1). Also when old mice of 21 months were investigated, we did not observe major alterations in their blood parameters (Table 1). In young mice, we only observed a mild increase in red cell distribution width (RDW), also called anisocytosis, which might indicate alterations in RBC production or turnover (Table 1). To determine RBC turnover rates, we compared the half-life of RBCs by measuring loss of biotinylated cells following in vivo labeling. No differences were observed between control and Noxa deficient mice, excluding the possibility that survival of mature erythrocytes is affected in the absence of Noxa (Fig. 4e). In addition, no alterations in EPO levels were observed (Fig. 4f), excluding an effect of Noxa ablation on endocrine cells.

**Table 1:** Characteristics of the RBC compartment in Noxa-deficient mice compared to syngeneic control mice

	8 weeks		21 months	
	WT	Noxa^−/−^	WT	Noxa^−/−^
Reticulocytes (%)	1.69 (0.32)	1.83 (0.64)	4.11 (0.30)	5.63 (0.78)
RBCs × 10^12^ L	9.09 (0.69)	8.61 (0.75)	8.13 (0.32)	8.00 (0.40)
Hemoglobin (mM)	9.73 (0.84)	9.00 (0.99)	4.10 (0.13)	4.08 (0.21)
Hematocrit (L/L)	53.90 (3.93)	53.77 (4.05)	54.95 (2.40)	50.34 (4.62)
RDW (%)	18.79 (1.19)	20.82 (3.05)^a^	19.3 (0.40)	19.35 (0.65)
MCV (fL)	58.31 (6.68)	60.12 (4.52)	ND	ND
MCH (fmol)	1.03 (0.04)	1.05 (0.03)^a^	1.00 (0.01)	1.03 (0.01)
MCHC (mM)	18.00 (2.39)	17.80 (1.59)	ND	ND
WBC × 10^8 ^L	8.66 (2.62)	12.16 (6.07)^a^	9.66 (3.02)	12.2 (4.10)

^a^Significant differences (*p* < 0.05)

Thus, even though Noxa mediates apoptosis of murine hematopoietic precursors under conditions of cytokine deprivation in vitro, its ablation has no discernible effects on the RBC compartment under homeostatic conditions.

### Noxa ablation results in enhanced acute stress-erythropoiesis

**Fig. 5:**
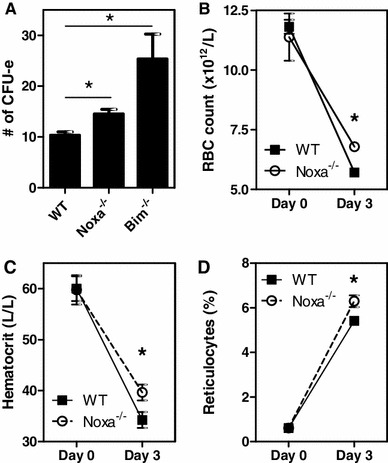
Noxa-deficiency provides partial protection against phenylhydrazine-induced acute haemolytic anemia. **a** 1 × 10^5^ Bone marrow cells of WT, Noxa^−/−^ or Bim^−/−^ were plated in semi-solid media following 32 h of culture in the absence of cytokines. After 3 days, CFU-e colonies were counted. The data show the number of colonies in cytokine deprived cultures. Data show a representative experiment of 4 independent experiments with 3 mice in each experiment. **b**–**d** WT and Noxa^−/−^ mice were injected i.p. with 60 mg/kg PHZ. 3 days after administration. **b** Red blood cell count, **c** hematocrit, and **d** reticulocyte count were determined. One of two independent experiments is shown, using 3–7 mice per experiment. *Error bars* represent SEM. *p < 0.05 (Student’s *t* test)

Considering the Noxa induction in erythroid progenitors and its regulatory role upon cytokine deprivation, the absence of an effect of Noxa-ablation on erythropoiesis appears unexpected. However, in homeostasis growth factors are amply available. The role of Noxa in erythropoiesis may thus only become apparent under conditions when expanding erythroid precursors are limited in their access to pro-survival cytokines. Indeed, when hematopoietic precursor cells were isolated from bone marrow and deprived from survival factors for 32 h before placing them in colony forming assays, Noxa-ablation provided a significant survival advantage to CFU-e progenitors compared to WT controls. In comparison, this survival advantage was increased considerably in cells deficient for Bim (Fig. 5a).

These findings suggested a role for Noxa when rapidly expanding erythroblasts must compete for the available growth factors, such as during acute anemia. Injection of phenylhydrazine (PHZ) in mice results in rapid haemolytic anemia, which is followed by robust proliferation of RBC precursors to compensate for RBC loss. Even though PHZ treatment results in an increase of serum EPO levels, this hormone is still rate-limiting for RBC expansion, as application of exogenous EPO results in increased RBC counts after PHZ induced anemia [26]. Noxa^−/−^ and WT mice were injected with PHZ [8, 27] to investigate recovery from acute anemia. After 3 days, hematocrit, RBC counts and reticulocyte counts were measured (Fig. 5b). RBC counts and hematocrit of WT mice were reduced to almost half the starting value. Hematocrit and RBC counts of Noxa^−/−^ mice were also considerably reduced, but showed significantly higher values than those of WT controls (Fig. 5b, c). Furthermore, reticulocyte numbers of Noxa-deficient mice were significantly higher following PHZ treatment compared to control mice (Fig. 5d). This indicates that Noxa deficiency results in enhanced RBC production, rather than protection against hemolysis.

Together, these data show that Noxa limits expansion of erythroid precursor cells under conditions of acute stress.

### Noxa ablation results in enhanced inflammation-induced stress-erythropoiesis

**Fig. 6:**
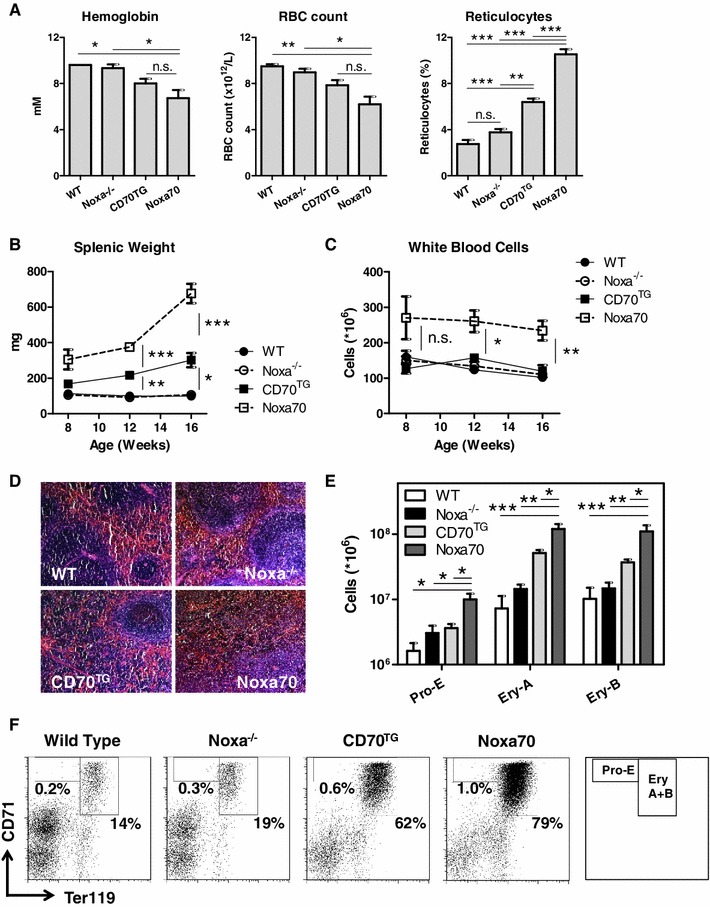
Increased expansion of erythroid progenitors in a model for inflammation-induced stress-erythropoiesis. Noxa^−/−^ mice were crossed with CD70^TG^ mice, to generate Noxa70 mice and were compared with WT, Noxa^−/−^ and CD70^TG^ animals. **a** Hematological blood parameters of 12 week old mice. **b** Splenic weight and **c** number of white blood cells in the spleens of mice at 8, 12 and 16 weeks of age. **d** HE staining of spleens of mice at 8 weeks of age (25× enlarged). **e** Absolute number of the erythroid progenitor populations Pro-E (CD71^+^Ter119^Dim^), Ery-A (CD71^+^Ter119^+^FSC^High^) and Ery-B (CD71^+^Ter119^+^FSC^Low^) in 8 week old mice. **f** Representative FACS plots of data shown in **e**. Gated is for nucleated cells (i.e. Ery-C and mature red blood cells are excluded). *Right most plot* shows which gates contain Erythroblast subsets. 3–4 animals are used per experiment. *Error bars* represent SEM. * *p* < 0.05, ** *p* < 0.005, *** *p* < 0.0005 (Student’s *t* test)

To corroborate our findings in a second system of stress-induced erythropoiesis, we made use of an available genetic model. CD70-transgenic (CD70^TG^) mice over-express the T cell co-stimulatory molecule CD70 on B cells and as a result have a high number of effector T cells in the bone marrow. Previously, we showed that IFNγ-production by these cells induces PU.1 in hematopoietic progenitors, which chronically impairs erythropoiesis in the bone marrow and causes severe anemia over time [28]. Since the spleen is the primary site of anemia-induced erythropoiesis in mice, CD70^TG^ mice have increased splenic stress-erythropoiesis [28]. We crossed Noxa-deficient animals with CD70^TG^ mice to generate Noxa70 mice [29], and investigated the effect of Noxa ablation on erythropoiesis in this system. When the blood of 12 week old mice was investigated, we observed clear symptoms of anemia in Noxa70 mice, as the RBC count and hemoglobin levels were significantly reduced compared to WT and Noxa^−/−^ controls (Fig. 6a; Table 2). However, no significant decrease was observed compared to CD70^TG^ mice. Strikingly, reticulocyte numbers were greatly increased in Noxa70 mice, indicating that these animals have increased RBC production. Indeed, whereas the number of white blood cells in the spleens of Noxa70 mice remained relatively constant (Fig. 6b, c), the splenic weight of these animals increased dramatically over time, suggesting increasingly extensive extramedullary erythropoiesis. This was confirmed by histological analysis of spleens, which revealed a greatly enlarged red pulpa in Noxa70 mice (Fig. 6d). Detailed analysis of cell subsets revealed that Noxa-deficiency in CD70^TG^ mice resulted in a dramatic increase of erythroblast precursor populations. This increase was observed both for early and late erythroid progenitors (Fig. 6e, f). Thus, also in a genetic model for chronic inflammation-induced stress, Noxa-deficiency results in an enhanced expansion of erythroid progenitors.

**Table 2:** Characteristics of the RBC compartment in Noxa70 mice compared to syngeneic control mice

	WT	Noxa^−/−^	CD70^TG^	Noxa70
Reticulocytes (%)	2.75 (0.40)	3.42 (0.34)	6.40 (0.37)	10.54 (0.57)
RBCs × 10^12^ L	9.50 (0.31)	8.97 (0.54)	7.84 (0.78)	6.21 (1.12)
Hemoglobin (mM)	9.60 (0.00)	9.33 (0.58)	8.00 (0.72)	6.73 (1.22)
Hematocrit (L/L)	46.83 (2.62)	47.93 (1.38)	49.53 (1.16)	38.8 (3.67)
RDW (%)	17.93 (0.50)	19.47 (0.51)	20.57 (0.42)	18.1 (0.78)
MCH (fmol)	1.69 (0.60)	1.73 (0.59)	1.69 (0.59)	1.82 (0.67)
WBC × 10^8^ L	9.40 (1.51)	9.87 (4.12)	3.53 (0.64)	5.33 (1.97)

In summary, Noxa controls expansion of erythroid precursors and RBC production in vivo under conditions of induced anemia.

## Discussion

Shortage of growth factors or nutrients triggers the mitochondrial apoptosis pathway resulting in rapid initiation of cell death, which is critically dependent on Bcl-2 family members. BH3-only proteins Bim and Puma have been demonstrated to play an important role in regulating the survival of hematopoietic cells [9, 20, 30–33]. Here we show that BH3-only protein Noxa regulates cell death of erythroid progenitors upon cytokine deprivation in vitro. In vivo Noxa regulates RBC production under conditions of anemic stress, when proper control of RBC expansion is required.

Noxa^−/−^ mice did not show elevated hematological blood parameters or increased erythroblast numbers in spleen and bone marrow under steady-state conditions, despite its induction during erythroid differentiation. In models of induced anemia, on the other hand, Noxa-deficiency promoted erythroblast expansion and enhanced recovery of hematocrit levels. Also when hematopoietic precursors were deprived of cytokines in vitro, Noxa mediated cell death of these cells. These findings indicate that apoptotic regulation of RBC production mediated by Noxa depends on the cellular demand of available vital cytokines. When expanding erythroblasts compete for limiting amounts of resources, which is particularly strong during stress-erythropoiesis, the non-redundant role of this pro-apoptotic protein became apparent. These observations are strikingly similar to our findings in lymphocytes. Noxa^−/−^ mice have no alterations in their T or B cell compartment under homeostatic conditions, when the amount of available pro-survival cytokines is abundant. In contrast, when these cells are activated, Noxa is induced and cells have to compete for the limiting amount of available nutrients. In WT mice, low-affinity cells have a competitive disadvantage and undergo Noxa-mediated apoptosis. In Noxa-deficient mice, these cells are allowed to survive and impact the affinity of T and B cell responses [14–16, 34]. Taken together, these findings indicate that Noxa-mediated apoptosis represents a general regulatory mechanism of highly expanding hematopoietic cells, that prevents expansion beyond the number of cells that can be sustained by the available cytokines.

The large number of EPO-regulated survival pathways that have been identified over the years [1, 35] may represent redundancy, but may also indicate separate functions at different biological stages. Noxa-deficiency rescued cytokine-deprived RBC precursors, but less so than Bim-deficiency (Fig. 5a). This difference in effect corresponds with the death-inducing potential of BH3-only proteins. Bim is considered a strong-inducer of apoptosis, since it interacts with all Bcl-2 family members and is able to induce cell death on its own [36, 37]. Noxa, on the other hand only interacts with Mcl-1 and A1 and its over-expression only increases sensitivity to pro-apoptotic stimuli [36, 37]. It appears that the death-inducing potential of these molecules is used to accomplish different levels of control over RBC expansion. Even though erythroid lineage commitment results in induction of both Bim and Noxa, only the former is required to regulate erythroblast expansion under homeostatic conditions [38]. However, under conditions of stress, when various activating stimuli induce rigorous proliferation in RBC precursors, Noxa aids Bim in preventing cellular increase beyond the number that can be sustained by the available amount of pro-survival factors. Thus, Noxa-mediated control of erythroblast expansion represents an additional layer of regulation for RBC expansion which only operates during stress-erythropoiesis, when Bim-mediated control is insufficient.

In summary, Noxa and its antagonist Mcl-1 are part of an important component of the mechanisms to re-establish RBC numbers upon blood loss and modulation of this pathway could be envisaged in therapeutic options for treatment of anemia.
